# Discussing racism in healthcare: A qualitative study of reflections by graduate nursing students

**DOI:** 10.1002/nop2.1619

**Published:** 2023-01-24

**Authors:** Elzana Odzakovic, Karina Huus, Beth Maina Ahlberg, Hannah Bradby, Sarah Hamed, Suruchi Thaper‐Björkert, Maria Björk

**Affiliations:** ^1^ Department of Nursing, School of Health and Welfare Jönköping University Jönköping Sweden; ^2^ CHILD Research Group, School of Health and Welfare Jönköping University Jönköping Sweden; ^3^ Department of Sociology Uppsala University Uppsala Sweden; ^4^ Skaraborg Institute for Research and Development Skövde Sweden; ^5^ Department of Government Uppsala University Uppsala Sweden

**Keywords:** graduate nursing education, healthcare, knowledge, nurse, qualitative study, racism

## Abstract

**Aim:**

The aim is to illustrate and analyse reflections from graduate nursing students over their experience of discussing racism in healthcare in an educational intervention.

**Design:**

A qualitative, descriptive design was adopted.

**Methods:**

Data were collected through written reflections and analysed through content analysis. In total, 81 students participated in the intervention; 39 paediatric and 42 public health care nursing students. Of those, 27 participants gave consent to have their written reflections included in the study.

**Results:**

Three main categories were developed in the content analysis of student reflections: (a) the implicit embeddedness of racism in healthcare organization; (b) the effect of racism on interactions with patients; and (c) a growing awareness of one's own understanding of racism. This study indicates that student nurses discussed racism as relevant to understanding good clinical practice for the benefit of patients and work‐based wellbeing of staff. This recognition of the organizational nature of racism warrants nursing leaders and managers to include racism as a social determinant of health in the undergraduate and graduate curricula to educate the next generation of nursing about racism.

## 1 INTRODUCTION

Racism, like other forms of unwarranted discrimination, whether involving staff, patients or both, damages the quality of care for patients and the quality of working environment for staff and the likelihood that people access care in the first place (Paradies et al., 2015). Racism persists even in societies that view themselves as colour blind (Bonilla‐Silva, 2014) and is a problem in Europe (Goldberg, 2006) as elsewhere. In this study, we define racism as a structural, historical and dynamic system of social organization based on a hierarchal arrangement of groups of people in racialized categories (Bonilla‐Silva, 1997), in which public policies, institutional practices, cultural representation, and social norms work in various ways to reinforce and perpetuate racial group inequity (The Aspen Institute, 2016). Racism's structural nature implies that it is embedded within social, economic and political networks and has material consequences that perpetuate racial inequalities (Omi & Winant, 2014).

Racism is prevalent in healthcare (Hamed et al., 2020) as much as in other institutions in society despite strong expectations that medical staff should behave ethically and with respect towards their patients. Racial inequalities in the process of delivering, receiving and accessing healthcare are documented across various national contexts (Hamed et al., 2022). Ethnic minorities across national settings report experiences of overt and covert racism by staff in healthcare such as dismissal of health conditions and symptoms, and disrespectful and rude treatment by staff (Sim et al., 2021). Ethnic minorities who experience racism have higher odds of reporting lower trust in healthcare and delay seeking care even when needed (Wamala et al., 2007). Staff, particularly ethnic majority staff, may exhibit implicit racial bias in favour of ethnic majority groups (Maina et al., 2018). Implicit racial bias is associated with inequalities in treatment choices in regard to pain management, maternal care, diabetes care, end of life care and other treatment choices (Hamed et al., 2022). Ethnic minority staff also experience racism from both patients and other staff (Fowler, 2020; Stevens et al., 2012) and report lack of organizational support and space to discuss racism which results in stress and emotional depletion (Ahlberg et al., 2022).

Discussions around racism in healthcare settings are difficult because racism is a topic that is seen as off‐limits in professional settings, despite evidence of clinical interactions, diagnosis and treatment choices being influenced by implicit racial bias (FitzGerald & Hurst, 2017). With regard to nursing education, racism has rarely been discussed among graduate students (Hall & Fields, 2015; Nairn et al., 2012). Instead, professional discussions among nurses have often focused on cultural competency in nursing (Bohman & Borglin, 2014) with little or no mention of racism. A study by Holland (2015), on anti‐racist teaching among nurses in the USA has shown that nurses found it difficult to speak about racism, let alone address it in teaching. Moreover, the majority of articles exploring anti‐racist medical training were conducted among USA medical students (Adelekun et al., 2019; Bright & Nokes, 2019). In contrast, little or no attention has been given to issues of racism, racist policies, or institutional racism, in nursing education in European settings. Since racism has an impact on the everyday lives of patients and staff (Iheduru‐Anderson et al., 2020), it is paramount to initiate a discussion about racism in healthcare and how to undo some of its effects, especially in a European setting where such discussions are rare. In this article, we describe on the outcome of a participatory action approach (Bradby et al., 2019) to identify, analyse, address and tackle racism in healthcare in Sweden.

## 2 AIM

This particular study aims to illustrate and analyse reflections from graduate nursing students over their experience of discussing racism in healthcare in an educational intervention. Before we outline our findings, we will describe the theoretical departure point that explains the context of the study, that is, postracialism as the prevailing ideology in the Northern European context, in particular Sweden, where this study was conducted.

### 2.1 Theoretical departure point

The idea of society in the Nordic region as exceptionally postracial has been widespread following World War II when ‘race’ was removed from official legal and policy documents (MacMaster, 2017). The removal of ‘race’ from official documents was in line with the UNESCO (United Nations Educational, Scientific and Cultural Organization) statement on ‘race’ issued first in 1950, which declared that ‘race’ is a social construct and rejected any scientific idea that perceives ‘race’ as a scientific or biological category (Shapiro, 1952). The preamble of the UNESCO constitution named racism as one of the ‘social evils’ of the world (Shapiro, 1952) and signified an important turning point, at least in regard to ‘race science’ (MacMaster, 2017). In many European settings, the rejection of scientific racism was largely based on ‘racial eliminativism’ (Kelly et al., 2010). The logic of this ‘eliminativism’ pertains to the following: First, ‘races’ were seen as a biological and scientific reality. Second, this view resulted in immense violence on European soil, which materialized in World War II. Third, as evidence emerged disposing of the biological nature of this social stratification, eradication of the category of ‘race’ was seen as essential since viewing the world in racial terms is inherently racist. Finally, by eliminating the notion of ‘race’ from legal and political discourse, the ‘eliminativist’ view concludes that racism has also eliminated given rise to a so‐called postracial society (Goldberg, 2006). This ‘eliminativist’ view has been adopted view in most Western and Northern European countries.

The fallacy of the postracial society is hence based on the (mistaken) notion that racism is a product of ‘race’ rather than a historical political system connected to colonial subjugation and oppression of various minority groups within and without Europe (Omi & Winant, 2014). The problem of the postracial illusion is that it seems to forget the history of racism, turns a blind eye to its contemporary formations and ongoing racialisation processes, and subsequently suggests that racism is an act of individual bias or prejudice (Bonilla‐Silva, 2009). According to Cho (2008), postracialism employs an idea of racial progress that entails that racism needs not to be addressed since society has achieved ‘historical accomplishment, or transcended racial divisions of past generations’ (p. 1601). Hence, if racism is dismissed as an individual bias rather than a structural system, and if racism is masked behind an idea of society as supposedly postracial, it becomes easy or common to regard antiracism as the inherent characteristic of European institutions (Lentin, 2008).

The Nordic region's sense of identity as exceptionally postracial (Keskinen, 2009) and where healthcare is embedded in neoliberal regimes, precludes a legitimate political position from which racism can be addressed critically, and makes research and discussions on racism difficult to undertake (Ahlberg et al., 2019). A postracial identity has thus shaped the Swedish society, evidence of racism notwithstanding (Lundström & Höjer, 2021).

## 3 METHODS

### 3.1 Design

A qualitative, descriptive design (Polit & Beck, 2021) for early testing of an intervention was used. Data were generated through written reflections (Jasper, 2005) from graduate nursing students after taking part in an educational intervention focusing on racism in healthcare. The data were subject to content analysis (Elo & Kyngäs, 2008).

### 3.2 Setting

The study was carried out at a university college in southeast Sweden. In Sweden, the training of specialist nurses is funded by the state. Students from two graduate nursing programmes—paediatric and public health—participated in the intervention. The specialist programmes comprise both theoretical and clinical modules which lead to a Master's degree in nursing. In the programme, all graduate nursing students are offered a university course, referred to as a module in what follows, called ‘Health Promotion in Nursing among Children and Adolescents’ (7.5 ECTS credits), where graduate nursing students get the opportunity to discuss and develop their knowledge about racism in healthcare in their role as specialist nurses. The study took place during the students' first semester.

### 3.3 Intervention focusing on racism in healthcare

The educational intervention, through the module, was mandatory and developed by the study authors representing two universities in Sweden. Four of the authors (BM, HB, SH, ST‐B) from one university in Sweden conducted primary research on racism in healthcare, that included semi‐structured interviews with 58 healthcare employees from a range of geographical and professional backgrounds, and including ethnic majority and ethnic minority Swedes (Bradby et al., 2019). All authors from both universities analysed the primary research on racism in healthcare to prepare for the educational intervention. The interview material was used to construct vignettes (Hughes, 1998) for use in the module on which this paper is based. The module required graduate nursing students to listen to a recorded lecture focused on racism in healthcare and read a scientific paper on racism in healthcare (Hamed et al., 2020), prior to participation in a seminar with a teacher‐moderated discussion of evidence‐based vignettes, drawing on the lecture, article and on students' own experience. The students were not offered a singular definition of racism, rather the lecture and the paper described ways that racism might manifest and why racism is not readily discussed in Sweden and in healthcare (Bradby et al., 2021). Students used the terminology that they felt was most suitable (Bradby et al., 2021).

The seminars were conducted in groups consisting of 8–9 students drawn from both the public health and the paediatric programmes. The seminars were conducted digitally via Zoom due to the COVID‐19 pandemic. At the beginning of each seminar, the students were given two vignettes, illustrating racism in healthcare. These evidence‐based vignettes were developed by the authors (HB and SH) from research interviews, to describe instances that were perceived as potentially racist.

The vignettes were shared with the students in a stepwise fashion in written and spoken form, with discussion questions interspersed with the delivery of further details about the case. All students were shown the same vignettes. This format resulted in a guided conversation with and between student healthcare professionals, supported by a constructive teacher‐led discussion about the experience of and opinions regarding racism in healthcare settings (Bradby et al., 2021).

### 3.4 Participants

In total, 81 students participated in the intervention; 39 paediatric and 42 public healthcare nursing students. Of the 81 students, 27 students from a range of geographical areas (small towns and big cities) and non‐migrant Swedes, and minority groups of migrant background, gave their written consent to have their written reflections included in this study. Most of the students were female. All these students were registered nurses with at least 1 year of clinical experience. Given the small sample size and due to confidentiality issues, further characteristics of individual participants, will not be described.

### 3.5 Data collection

The students received both oral and written information about the study, prior to the intervention. All students handed in a written reflection of no more than 300 words after taking part in the seminar and 27 students gave their consent for their written reflections to be included in this study. The students were asked to reflect on whether they had discussed racism in healthcare settings with their colleagues and if so, how these discussions had played out; and if not, why such discussion had not taken place. Furthermore, they were asked to reflect on the most interesting and surprising things that had been highlighted during the seminars, whether they had gained understanding about racism, and the extent to which they felt able to discuss racism in healthcare settings in their future work.

### 3.6 Analysis

The written reflections were analysed based on the principles of qualitative content analysis (Elo & Kyngäs, 2008). All authors of this study were involved in the analysis and validated the categories. An inductive approach was applied to the reflections to identify categories, phrases and keywords that represent the phenomena being described. This process included three steps: open coding, creating categories and abstraction analysis (Elo & Kyngäs, 2008) to analyse the data. First, the data were re‐read, and notes and headings were written in the margins to describe the content. Second, after open coding, the data were grouped into categories. A list of categories was developed depending on how they were related or belonged to a group, and sub‐categories were identified. Definitions for these categories were developed and named by content‐specific words. Third, to achieve confirmability of the findings, the researchers individually review the data, then developed the categories and came to agreement of the categories through consensus (Elo et al., 2014). Lastly, to ensure credibility, an appropriate data collection method was selected and member checking within the research group (Elo et al., 2014).

### 3.7 Ethics statement

This study was formally approved by the Uppsala Regional Ethics Board (Dnr 2018/201) and funded by the Swedish Research Council (Dnr: 2016‐04078). Participation was voluntary, and the participants were assured of confidentiality. They were given both oral and written information and had time to ask questions and reflect on their participation before giving informed consent. They were assured that their participation or non‐participation would not affect their course grades. To protect the confidentiality of the participants, we have given the students pseudonyms which are largely gender neutral, to avoid identifying the small number of men.

## 4 FINDINGS

**FIGURE 1:**
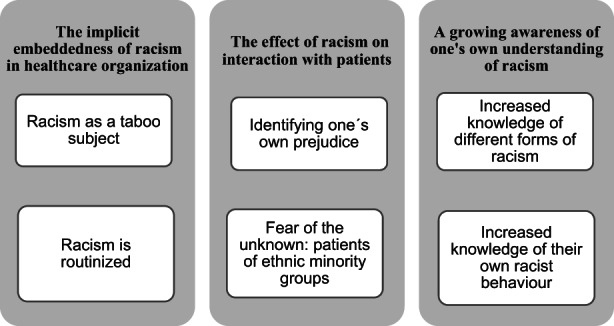
Presentation of the categories and sub‐categories

The analysis of the written reflections identified three main categories: (a) the implicit embeddedness of racism in healthcare organization; (b) the effect of racism on interactions with patients; and (c) a growing awareness of one's own understanding of racism. Within the main categories, six sub‐categories were identified as shown in Figure 1.

### 4.1 The implicit embeddedness of racism in healthcare organizations

This first category exposes how racism is embedded in healthcare organizations. Students said that racism is hidden in healthcare organizations and is not discussed at any level of the organizational hierarchy. Racism was said to be a taboo and loaded issue, with students reporting that racism often occurred without any subsequent reflection or discussion on the matter reflecting the idea of Sweden as a supposedly postracial society. This lack of discussion was attributed to the taboo surrounding racism, which also contributed to it becoming routinized in healthcare. These two features of racism are discussed in the next two sub‐sections.

#### 4.1.1 Racism as a taboo subject

According to the students, not everyone had experienced racism and neither was it openly discussed among colleagues. Opportunities to discuss racism at meetings or during daily work were rare. A major reason why racism was not discussed at their workplace is because it is a “sensitive” and “taboo” subject. As two students, Kim and Ariel, stated:

At work, we have not discussed racism as far as I can remember. I think that the main reason it is not discussed is because it is a sensitive subject and you don't want to be labelled as a racist. (Kim)

I think this is a taboo subject that no one really dares to bring up. Somehow, we pretend that we have not heard anything and just keep going. It surprised me that we have all encountered this, but that we almost never bring it up for discussion. (Ariel)

Beyond the fear of being labelled a racist, students also reported apprehension at the organizational level about discussing this type of topic. The organizational apprehension implied a range of negative emotions at the thought of discussing racism such that students turning a blind eye to the fact that racism even exists. This type of response means that racism is silenced and normalized and thus rendered invisible in healthcare organization. Students said that this culture of silence is, embedded in healthcare and results in an environment of apprehension where few dare to highlight and discuss racism. Even if they have been exposed to it, few dare or have the energy to report racism to management, as indicated by Simone and Tove:

The reasons why racism is not discussed is because one closes one's eyes to these problems in healthcare. Unfortunately, when someone has been exposed to racist opinions, many people probably do not dare or have the strength to go ahead and report it. (Simone)

I have also been with patients who do not want to meet a doctor who is not from the ethnic majority, but this is something that has not been raised and discussed in our organization. (Tove)

It was stressed that the invisibility of racism in healthcare should be addressed so as to improve the working environment. According to the students, racism in healthcare should no longer be ignored or silenced; they embraced the need for an action plan at the organizational level to deal with racism towards healthcare staff and patients thereby improving the working environment for both providers and users of healthcare.

#### 4.1.2 Racism is routinized

A common perception among students was that racism is embedded in healthcare routines, particularly when it comes to patients from minority ethnic groups. Students described how bias based on patients' ethnicity occurred when patients' names were identified as ‘non‐Swedish’ or their appearance was associated with a racial or ethnic minority. These actions were often routine without reflection on whether they harboured a racist effect. Vanja, one of the students who had worked in primary healthcare, describe the experience of everyday routinized racism:

Racism can become a routine, for example, when you book patients who you think you need an interpreter just because of his/her name displayed on the computer. It is discrimination. (Vanja)

Other students also emphasized the importance of reviewing routines and culture in healthcare. The underlying problem was said to be recurrent racism that is reproduced without reflecting on its impact on patients and the risk of patients receiving differential treatment because of their ethnicity. One example, was patients from ethnic minority groups, who consulted over problems of pain, but who were subsequently ignored. Robin described how there was a perception that patients from ethnic minority groups had a different expression of pain compared to native Swedes and were assumed to exaggerate their symptoms:

It was common that patients of ethnic minority groups with pain were neglected to a greater extent. That these patients would express pain differently than native Swedes. (Robin)

Students' reflections suggest that racism is embedded in healthcare routines and the organizational contexts in which healthcare is provided.

### 4.2 The effect of racism on interactions with patients

This category presents students' experiences of encountering patients in different situations involving racism. The students shared a fear of the unknown in meeting patients, which is illustrated in the next two sub‐categories to be presented: first, identifying one's own prejudice and second, fear of the unknown: patients of foreign background leading to fear of the unknown.

#### 4.2.1 Identifying one's own prejudice

The topic of appearance that identified a racial or ethnic identity was said to be considered only when taking care of patients from ethnic minority groups, but not with regard to racism. For instance, students reported a sense among staff that patients and relatives would not listen to healthcare advice and would refuse to follow medical advice, simply due to their foreign origin. Tintin, who worked in paediatric care, described having been in several meetings where colleagues cast doubt on the parenting ability of mothers of ethnic minority group:

At one point, I told some colleagues who spoke inappropriately about a mother from an ethnic minority group, that they assumed that she would be a certain way just because she was a foreigner. (Tintin)

Students said that silent and unconscious racism is ongoing in consultations. Students reported that even small racist acts could be significant to patients, because they may be deprioritized and hence not receive adequate care. Students believed that it is difficult to change their colleagues' attitudes and prejudices towards patients of Swedish minority ethnic groups and describe this prejudice as often rooted in a fear of meeting patients who were seen as foreign.

#### 4.2.2 Fear of the unknown: patients from of ethnic minority groups

Some students said that patients' foreign background leads to a fear of the unknown, which affects the patient encounter. Having limited knowledge of another (minority) culture gave rise to a fear of saying something that would be misinterpreted. Students said that they are often afraid to ask the patient if they do not understand something that may have an impact on their treatment, in case the students are branded as racist. According to the students, the main reason for this lack of communication was a fear of the unknown. Juno, who worked in primary healthcare, expressed what the lack of discussion and communication can lead to:

It felt as if the discussion was largely based on the fact of misunderstandings and meetings that took place when things did not go well. It is about the lack of communication, feelings of vulnerability and fear of the unknown. (Juno)

### 4.3 A growing awareness of one's own understanding of racism

In this category, students described how important the seminar about racism was for them. They gained insight into what racism can look like in different situations, viewing the seminar as an eye‐opener. Students perceived that they gained knowledge of different forms of racism and an increased awareness of how their own behaviour could be racist or have racist effects.

#### 4.3.1 Increased knowledge of different forms of racism

In all the written reflections, students mentioned that they gained knowledge of the structural and individual aspects of racism. They had been unaware that racism can exist at different organizational levels in healthcare but also in general in Sweden. For instance, Jasmin and Helle, who both have several years of nursing experience, reported:

I have not previously thought about the fact that discrimination and consequently racism can be based on a structural level, which is important to include in any improvement work around the patients. (Jasmin)

It was important and rewarding to address structural racism and not just on a personal level. It is important to be aware of how it can affect different individuals and become more attentive and aware of what racism can look like. (Helle)

To understand racism as embedded in institutional structures was surprising for students, because they had not thought of racism as anything other than interactional, occurring between individuals. Organizational or structural racism had not been discussed in the workplace or with colleagues. Beyond the new awareness of structural racism, students also indicated they had not previously been aware of how their colleagues on the course experienced racism differently from themselves. Some discussion topics at the seminar were new for them and they would never have linked them to racism. This opened up their own understanding that racism was common for some healthcare staff who could be regularly exposed to racism by patients. Helfrid and Charlie shared their experiences about the seminar with us:

The seminar has given me more understanding that racism is common, especially among healthcare staff, and that they are exposed to racism everyday by patients. I did not think this was so widespread. (Helfrid)

The seminar gave me a lot to think about. but I mainly found out that there is a need for having action plans for how both staff and patients should proceed when they experience racism in healthcare. (Charlie)

In addition, the students expressed how they gained knowledge about different forms of racism; for example, when someone tries to justify certain care decisions that neglect the patient's own concerns, but then say that their actions were right and that they were not a racist. Students said that these understandings of different concepts of racism and discrimination could be taken back to their workplaces.

#### 4.3.2 Increased knowledge of their own racist behaviour

Students stated that they personally started to reflect on their own behaviour with regard to racism. Some reported becoming more aware of what their behaviour and actions could lead to. It also became easier to talk about racism with others because they were now more knowledgeable about it. Inge said:

Personally, I bring with me a greater awareness of racism, and how important it is to examine one's actions through new eyes so that I do not unconsciously contribute to structural racism. (Inge)

Knowledge of their own behaviour increased by reading the research article and reflecting on racism in the seminar, where they had an opportunity to engage in a group discussion. However, some students also reported that they were still not comfortable with discussing racism with their colleagues even though they gained knowledge; there were still obstacles in bringing up the subject with others because they themselves had not been exposed to racism. Love and Renée shared their experiences about talking about racism both personally and in healthcare:

It is difficult to understand what it is like to be exposed to racism and how it can be experienced; it is a fundamental right for everyone to be treated with dignity and respect. (Love)

I'm not very comfortable talking about racism because it is not directed at me. I believe that I have no interpretive precedence on what is and is not racism. (Renée)

Students reported that their own reluctance to discuss racism was characteristic of the nursing profession. As a nurse, you should give the same care to all regardless of the patient's country of origin and this vision of what a professional and caring nurse should aspire to, prevented reflection over behaviour that could have discriminatory effects. The reluctance to consider racism meant that students wanted their manager or someone at their current workplaces to raise the question of racism because it felt like such a taboo subject for many. According to the students, group discussions and reflections could remove some of the worries about being seen to be a racist or saying something wrong that may hurt patients or colleagues.

## 5 DISCUSSION

The article analyses an educational intervention, built on a structured discussion of vignettes derived from research on undoing racism (Bradby et al., 2019), that resulted in reflective writing by graduate nursing students. To our knowledge, this is the first educational intervention on racism in healthcare in a Swedish medical setting. The exceptional image of Sweden as an anti‐racist country (Hübinette & Lundström, 2011) where racism is often minimized, even when evidence of racism exists, contributes to the silencing and routinization of racism. In their reflections, graduate nursing students not only confirm the silence around racism but they also indicate what needs to be done to change the silence. Previous studies in other settings also suggest that there is a silent racism in healthcare and that nursing students were expected to remain silent and accept the situation despite discriminatory behaviours (Ackerman‐Barger & Hummel, 2015; Ahlberg et al., 2019; Coleman, 2008; Holland, 2015; Iheduru‐Anderson et al., 2020). Therefore, a dialogue about different forms of racism needs to be developed in healthcare and nursing education, according to our participants. Nursing is considered a caring profession and nurses are educated and trained to give nursing care to all people regardless of race or ethnicity (Beard et al., 2020; Cooper Brathwaite et al., 2022). Nurses must therefore uphold the standards of care and ethical principles of nursing when providing care for all patients (International council of nurses, 2021). This implies the importance of not seeing patients from ethnic minority groups as being “problematic” or as “different from the norm”, but to see each individual person as unique and address their needs in order to receive an equitable and accessible care (Koschmann et al., 2020). Therefore, nursing education needs to be developed to address racism as a social determinant of health in the undergraduate and graduate curricula to educate the next generation of nursing (Brown et al., 2019; Lopez, 2020; Morley et al., 2020). By doing this, students get opportunities to develop their knowledge about racism in healthcare and to address fears that some expressed about patients from of ethnic minority groups (DiAngelo, 2012; Russell & Flynt Wallington, 2022). This is of importance as Mental Health of America (2020) found that racism causes stress in Black people, Indigenous people and people of colour, which resulted in depression, anxiety and post‐traumatic stress disorder (PSTD). Our study indicates that the students are forthcoming about their role as potential participants for change—a shift in ways of working within healthcare systems.

This study reveals student nurses' understandings of the silencing and routinization of racism. Students were engaged with the problem of understanding racism in healthcare settings, from staff and patient perspectives, and they appreciated that individual‐level interactions were not the only way that racism could occur, since organizational routines and practices were also relevant. While concepts such as structural racism were not universally embraced by our participants, and individual‐level fear of the unknown was expressed, racism was widely seen as an issue relevant for nurses' professional development. Therefore, it is important that graduate nursing students are introduced to what impact structural racism can have on patients and their own professional role (Coleman, 2008; Holland, 2015; Iheduru‐Anderson et al., 2020).

Healthcare managers need to understand how these processes play out across the entire healthcare organization in order (Baack & Fischer, 2011) to design interventions to dismantle systemic racism. This intervention study was one step towards this goal, demonstrating, as it does, that constructive discussion across a diverse group of student nurses, with a range of experience and opinions is both feasible and welcomed by those students. Confirmation of the feasibility of constructive discussion from some students does not imply wholesale agreement, since some students in this study had difficulty speaking about racism and preferred not to acknowledge the issue of racism by remaining silent. The lack of total agreement does not, however, undermine the warrant to develop anti‐racism approaches in nursing, on the grounds of equity and justice.

### 5.1 Implications for clinical practice and education

- Racism must be acknowledged as a process relevant for health and wellbeing by nursing leaders at all levels and be opened up for discussion among staff and students.
- Nursing leaders and managers should include racism as a social determinant of health in the undergraduate and graduate curricula to educate the next generation of nursing about racism.
- Provide workshops on racism which should be given in orientation programmes to new staff and permanent staff in academic and healthcare settings, so all staff gain an understand of the damaging effects of racism (depression, anxiety and post‐traumatic stress disorder [PSTD]).

### 5.2 Limitations

It should be acknowledged that the study has its limitation. The inclusion of only graduate nursing students in this research may represent a limitation of the study. The participants in this study are nurses in an on‐going postgraduate education in paediatric and public health, which means that we have not included other students from other educational streams. Further work should include a broader sample of participants in various educational settings as a variety of different groups of nursing students will give additional information based on their experiences. The proportion of the students that participated in the seminars and who then gave permission to have their written reflections analysed is one third (27 out of 81). Since we cannot subject the remaining 54 students' reflections to analysis, we cannot state the extent to which the views stated are characteristic of the whole group of 81 students.

## 6 CONCLUSION

The existing literature documents that racism as a topic is rarely discussed among graduate nursing students (Hall & Fields, 2015; Holland, 2015; Nairn et al., 2012) and the focus has mostly been on cultural competency in nursing (Bohman & Borglin, 2014). This study contributes to the existing literature by revealing the need for dialogue and action in nursing education and in healthcare about racism in healthcare. It is time to recognize the silent racism in healthcare and start to educate nurses about racism. This study has shown that graduate nursing students are willing and able to constructively reflect on how racism might affect their practice with one another. A development of this study would be to convene joint discussions where graduate nursing students, patients and healthcare staff work together and exchange their experiences of racism. Further studies should focus on this.

## AUTHOR CONTRIBUTIONS

Elzana Odzakovic designed the study, led the analysis and collected data and drafted the manuscript. Karina Huus was involved in designing the study and in the data analysis and drafting the manuscript. Beth Maina Ahlberg was involved in designing the study and in the data analysis and drafting the manuscript. Hannah Bradby was involved in designing the study, collecting data and drafted the manuscript. Sarah Hamed was involved in designing the study, collecting data and drafting the manuscript. Suruchi Thaper‐Björkert was involved in designing the study and drafting the manuscript. Maria Björk was involved in designing the study, collecting data and drafting the manuscript.

## FUNDING INFORMATION

Funding was provided by the Department of Health Sciences (Jönköping University), the Department of Sociology, the Department of Government (Uppsala University), Sweden and the Skaraborg Institute for Research and Development.

## CONFLICT OF INTEREST

The authors declare no conflicts of interest.

## ETHICAL APPROVAL

This study was formally approved by the Uppsala Regional Ethics Board (Dnr 2018/201) and funded by the Swedish Research Council (Dnr 2016‐04078). Participation was voluntary, and the participants were assured of confidentiality. They were given both oral and written information and had time to ask questions and reflect on their participation before giving informed consent. They were assured that their participation or non‐participation would not affect their course grades. To protect the confidentiality of the participants, we have given the students pseudonyms which are largely gender neutral, to avoid identifying the small number of men.

## Data Availability

The data that support the findings of this study are available from the corresponding author upon reasonable request.
